# Comparison of whole transcriptome sequencing of fresh, frozen, and formalin-fixed, paraffin-embedded cardiac tissue

**DOI:** 10.1371/journal.pone.0283159

**Published:** 2023-03-29

**Authors:** Stine Bøttcher Jacobsen, Jacob Tfelt-Hansen, Morten Holdgaard Smerup, Jeppe Dyrberg Andersen, Niels Morling

**Affiliations:** 1 Section of Forensic Genetics, Department of Forensic Medicine, Faculty of Health and Medical Sciences, University of Copenhagen, Copenhagen, Denmark; 2 Department of Cardiology, Rigshospitalet, Copenhagen University Hospital, Copenhagen, Denmark; 3 Department of Cardiothoracic Surgery, Rigshospitalet, Copenhagen University Hospital, Copenhagen, Denmark; Universita degli Studi di Salerno, ITALY

## Abstract

The use of fresh tissue for molecular studies is preferred but often impossible. Instead, frozen or formalin-fixed, paraffin-embedded (FFPE) tissues are widely used and constitute valuable resources for retrospective studies. We assessed the utility of cardiac tissue stored in different ways for gene expression analyses by whole transcriptome sequencing of paired fresh, frozen, and FFPE tissues. RNA extracted from FFPE was highly degraded. Sequencing of RNA from FFPE tissues yielded higher proportions of intronic and intergenic reads compared to RNA from fresh and frozen tissues. The global gene expression profiles varied with the storage conditions, particularly mitochondrial and long non-coding RNAs. However, we observed high correlations among protein-coding transcripts (ρ > 0.94) with the various storage conditions. We did not observe any significant storage effect on the allele-specific gene expression. However, FFPE had statistically significantly (p < 0.05) more discordant variant calls compared to fresh and frozen tissue. In conclusion, we found that frozen and FFPE tissues can be used for reliable gene expression analyses, provided that proper quality control is performed and caution regarding the technical variability is withheld.

## Introduction

The introduction of next-generation sequencing (NGS) has enabled large-scale genetic, epigenetic, and gene expression analyses. The potential use of high-throughput RNA-sequencing to uncover molecular mechanisms of human cardiac diseases has been acknowledged [1–5]. However, due to the tissue-specific nature of gene expression, a major limitation of RNA-sequencing in the investigation of cardiac diseases is the limited availability of cardiac tissue.

Formalin is one of the most widely used fixatives to preserve biological specimens in clinical and forensic histopathology laboratories [6]. Hence, vast repositories of formalin-fixed, paraffin-embedded (FFPE) tissue samples are found worldwide, representing an important resource for retrospective studies. The advantages of FFPE includes the possibility of long-term storage at room temperature and cross-linking of biomolecules, which preserves tissue morphology and thereby makes FFPE tissues suitable for immunohistochemical staining [7]. However, formalin fixation causes degradation of nucleic acids and induces sequence artefacts, which may limit the use of FFPE tissue for genetic, epigenetic, and gene expression analyses [8–10].

Several studies investigated the robustness of RNA-sequencing from FFPE tissue as well as the concordance between gene expression patterns in frozen and FFPE tissue [10–15]. Most studies reported that FFPE tissue is suitable for NGS-based gene expression analysis. However, certain genes and pathways seem to have increased susceptibility to the deleterious effects of the fixation process, such as pathways of oxidative stress, mitochondrial dysfunction, DNA/chromatin packaging, and nucleosome organization [11, 16]. In addition, it has been shown that sequencing of RNA from FFPE tissue generates increased numbers of off-target reads associated with intronic and intergenic regions, whereby transcript isoform analysis based on RNA from FFPE tissue may be challenging [11].

The use of frozen tissue for the sequencing of nucleic acids is considered the gold standard as fresh tissue is rarely available. However, to our knowledge, the correlation between gene expression profiles of RNA extracted from fresh and frozen tissues has not been reported.

In this study, we performed whole transcriptome sequencing (WTS) from paired fresh, frozen, and FFPE tissue to investigate the effect of tissue storage conditions on gene expression profiles. We assessed the RNA integrity number (RIN) and length of RNA fragments extracted from fresh, frozen, and FFPE tissue to evaluate the stability of RNA under various storage conditions. In addition, we investigated the alignment of WTS data to assess the proportion of off-target (intronic and intergenic) reads and the abundance of various RNA types. Furthermore, we compared the global gene expression patterns to determine the utility of tissues with different storage conditions for gene expression analysis. Lastly, we compared genotypes derived from RNA sequencing reads with DNA genotypes for cardiac-disease relevant genes to investigate the concordance between RNA and DNA variants for the different storage conditions. This is especially of relevance for studies of allele-specific gene expression (ASE) as discordant variant calls between heterozygous DNA variants and RNA variants may indicate allele-specific gene expression.

## Materials and methods

### Ethics statement

The study conformed to the Declaration of Helsinki and was approved by the Committees of Health Research Ethics in the Capital Region of Denmark (H-20039524). The biobank, where the samples are stored, is registered at the University of Copenhagen’s joint records of processing of personal data in research projects and biobanks (514-0528/20-3000), and it complies with the rules of the General Data Protection Regulation (Regulation (EU) 2016/679). Informed written consent was collected from all individuals. Patient data were pseudonymised.

### Study population and tissue collection

**Fig 1:**
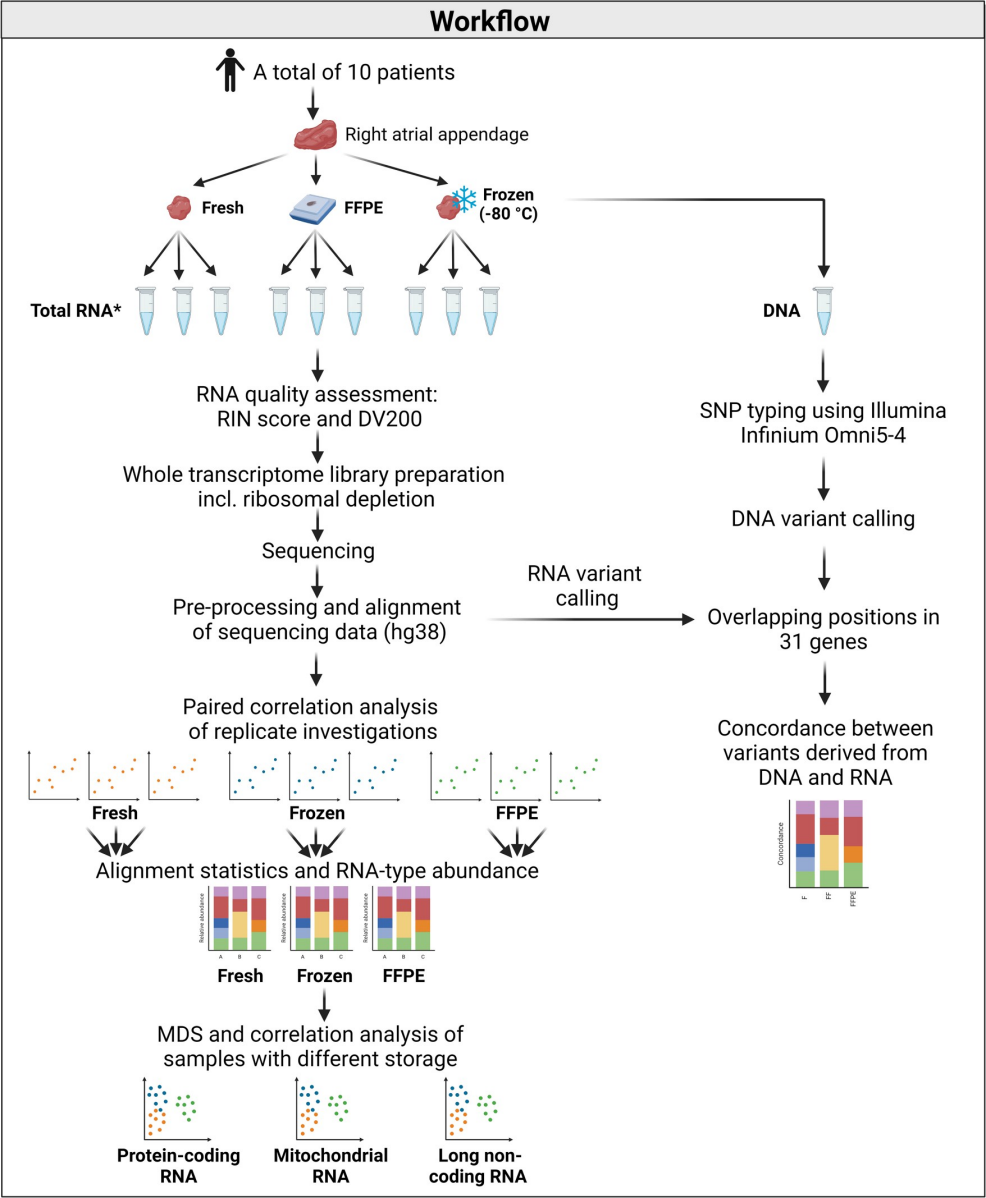
Graphical representation of the workflow. *Only one RNA extraction from each storage condition from patient 5. Abbreviations: DV200: Percentage of RNA fragments > 200 nucleotides, FFPE: Formalin-fixed, paraffin-embedded, RIN: RNA integrity number, SNP = Single nucleotide polymorphism. Created with Biorender.com.

Biopsies of human right atrial appendage (RAA) tissue were collected from 10 patients that underwent scheduled cardiac surgery at Rigshospitalet, Copenhagen University Hospital, Copenhagen, Denmark. Descriptive data on the patients included in the study is presented in Table 1. All RAA samples were divided into three pieces: one was used for nucleic acid extraction immediately after tissue collection (fresh tissue), one was frozen at -80°C (frozen tissue), and the last piece was fixed in formalin and embedded in paraffin (FFPE tissue). The median time from tissue collection to nucleic acid extraction/freezing/fixation was 27 minutes (range: 22–41 minutes). A graphical representation of the workflow can be found in Fig 1.

**Table 1:** Descriptive characteristics of the study population.

Characteristics	n = 10
Male sex, n (%)	9 (90%)
Median age at time of tissue collection, years (range)	66.5 (50–79)
Type of surgery performed*, n (%)	
CABG	7 (70%)
Valve repair/replacement	4 (40%)
Ascending aorta aneurysm	1 (10%)
History of atrial fibrillation, n (%)	
Before surgery	2 (20%)
During surgery	0 (0%)
After surgery	5 (50%)

* Two patients underwent both coronary artery bypass graft (CABG) and valve repair/replacement surgery.

### Formalin fixation and paraffin embedding

RAA tissues were fixed in 4% buffered formaldehyde using the BiopSafe Biopsy Sample System (BiopSafe, Denmark). Fixation times ranged from 23–97 hours (median: 62 hours). Tissue dehydration and paraffin- treatment was performed on a Tissue-Tek VIP 6 AI (Sakura Finetek Europe, the Netherlands) and included the following incubations: 1 x 4% buffered formaldehyde for 60 min., 6 x EtOH for 90 min. with increasing concentrations of EtOH, 2 x Histolab Clear (Histolab Products AB, Sweden) for 60 min., 1 x Histolab Clear for 120 min., and 4 x Paraffin for 80 min. Lastly, tissues were embedded in paraffin.

### RNA extraction

Total RNA from fresh and frozen tissue was extracted using the RNeasy Fibrous Tissue Mini Kit (Qiagen, Germany) following the manufacturer’s instructions. Approximately 30 mg of tissue was used as input, and the tissue was homogenised for 2 x 2 min at 20 Hz using the TissueLyser II (Qiagen, Germany). On-column DNase treatment was performed. Frozen tissue samples were stored at -80°C for a median time of 27.5 days (range: 20–40 days) before RNA extraction.

Total RNA from FFPE tissue was extracted using the RNeasy FFPE Kit (Qiagen, Germany) following the manufacturer’s recommendations. A total of 2 x 20 μm FFPE sections (tissue ~ 7 x 7 mm) were used per extraction. Paraffin was removed using xylene followed by a wash in 96–100% ethanol. On-column DNase treatment was performed. FFPE blocks were stored at room temperature for a median time of 29 days (range: 20–42 days) before RNA extraction.

The RNA extractions from nine of 10 patients were performed in triplicate. Due to the limited amount of tissue from one individual, a single RNA extraction from each storage condition was performed. The quantity of the extracted RNA was assessed using the Qubit RNA HS Assay (Invitrogen, USA). The quality of the RNA was assessed in terms of the RIN and the percentage of RNA fragments > 200 nt (DV200) using the Bioanalyzer RNA 6000 pico assay with the 2100 Bioanalyzer system (Agilent, USA). The 2100 Bioanalyzer system (Agilent, USA) could not calculate the RIN of six RNA extractions.

### Whole transcriptome sequencing

Whole transcriptome library preparation was performed using the SMARTer® Stranded Total RNA-Seq Kit v3 –Pico Input Mammalian (Takara Bio Europe, France) according to the manufacturer’s instructions. Briefly, 10 ng total RNA was fragmented, cDNA synthesis was performed using random N6 primers, multiplexing indices and adapters were added, and ribosomal cDNA depletion was performed. Due to the fragmented nature of the RNA extracted from FFPE tissue, the fragmentation step was omitted in the library preparation of RNA from FFPE tissues. AMPure XP beads (Beckman Coulter, USA) were used instead of the recommended NucleoMag NGS Clean-up and Size Select beads (Macherey-Nagel, Germany). The qualities of the cDNA libraries were assessed using the Bioanalyzer High Sensitivity DNA assay with the 2100 Bioanalyzer system (Agilent, USA). The quantities of the cDNA libraries were assessed using the 7500 Real-Time PCR System (Applied Biosystems, USA) with the KAPA Library Quantification Kits–Complete kit (ABI Prism) (Kapa Biosystems, USA). Paired-end sequencing (2 x 100 bp) was performed on a NovaSeq 6000 instrument using the NovaSeq 6000 S1 Reagent Kit v1.5 (Illumina, USA).

### Alignment and normalisation

BCL-files were converted to FASTQ-files using *bcl2fastq2* (Illumina, USA), and the sequencing quality was assessed using *FastQC* [17]. Adapter sequences, consecutive stretches of low-quality bases (Q<30) at the 5’ and 3’ termini, and reads shorter than 20 bp were removed using *AdapterRemoval* version 2.3.2 [18]. The reads were aligned using *STAR* version 2.5.3a [19] with default settings except for two parameters. Reads aligned to multiple loci were classified as unmapped (—outFilterMultimapNmax 1), and the prediction of unannotated splice junctions was disabled (—alignIntronMax 1). Alignment statistics and gene count files were generated with *STAR* based on the human genome assembly GRCh38.p13 (hg38) and FASTA- and GTF-files from the GENCODE consortium release 33 [20]. Subsets of protein-coding RNAs, mitochondrial RNAs (mtRNAs), and long non-coding RNAs (lncRNAs) were generated based on gene annotations in the GENCODE consortium release 33 [20]. Graphics of alignment statistics were created using the *ggplot2*-package version 3.3.5 [21] in *R* version 4.0.2 [22].

Gene counts from replicate investigations were summed before normalisation. To account for differences in sequencing depth, gene counts from the protein-coding, mtRNA, and lncRNA subsets were normalised according to the total read count within the subset. Gene counts were reported as moderated log_2_(counts-per-million (CPM)) using the *CPM(x*, *log = TRUE)*-function in the *edgeR*-package version 3.32.0 [23] in *R*. The moderation means that a small number, proportional to the sequencing depth, was added to all values to avoid taking the log of zero.

### Comparison of global gene expression

Multidimensional scaling (MDS) plots of the protein-coding, mtRNA, and lncRNA subsets were generated using the *plotMDS()*-function in the *limma*-package version 3.46.0 [24] in *R*. Distances in the MDS-plots correspond to the leading log_2_(fold-change (FC)), which describes the root-mean-square average of the log_2_(FC) for genes best distinguishing each pair of RNA samples [24]. The aesthetics of the MDS-plots were improved using the *ggplot2*-package.

Spearman’s correlation analyses of intra-individual gene expression profiles of the protein-coding, mtRNA, and lncRNA subsets from fresh, frozen, and FFPE cardiac tissue were performed and visualised using the *GGally*-package version 2.1.2 and *ggplot2*-package in *R*.

### RNA variant calling

Adapter trimming using *AdapterRemoval* version 2.3.2 [18] and alignment using *STAR* version 2.5.3a [19] were carried out as described for the gene expression analysis. The subsequent analysis followed the Genome Analysis ToolKit (GATK)s Best Practices Workflow for RNAseq short variant discovery, March 2022 (https://gatk.broadinstitute.org/hc/en-us/articles/360035531192-RNAseq-short-variant-discovery-SNPs-Indels-). After alignment, duplicate reads were marked using *MarkDuplicates* from Picard (http://broadinstitute.github.io/picard/). Hereafter, *GATK* version 4.2.0 *SplitNCigarReads* was used to split reads with N in the CIGAR into multiple supplementary alignments. Because *STAR* is an RNA aligner, the alignments spanning introns had to be reformatted before variant calling using *GATK*. Base quality recalibration was carried out using *GATK* version 4.2.0 *BaseRecalibrator* and *GATK* version 4.2.0 *ApplyBQSR* following recommended settings. Variant calling was performed using the *GATK* version 4.2.0 *HaplotypeCaller* with recommended settings.

### DNA extraction

Due to the limited amounts of fresh tissues, frozen tissues were used for DNA extraction. The DNA extraction was carried out using the DNeasy Blood & Tissue Kit (Qiagen, Germany) following the manufacturer’s recommendations for purification of total DNA from tissue.

### SNP typing using Illumina Infinium Omni5-4

Samples were analysed using the Illumina Infinium Omni5-4 BeadChip kit (Illumina, USA) following the manufacturer’s recommendations with 400 ng DNA input. BeadChips were scanned using the iScan™ system (Illumina, USA) following the manufacturer’s recommendations. Genotyping was carried out using Illumina GenomeStudio with default settings.

### Concordance between RNA and DNA variants for cardiac disease-related genes

RNA sequencing variants were retrieved for OMNI5-4 positions within the exon regions for genes involved in cardiac disorders from the American College of Medical Genetics and Genomics (ACMG) secondary findings (SF) v3.0 list [25] or genes implicated in atrial fibrillation [26]. To ensure the quality of the detected DNA and RNA variants, only variants with read depth ≥ 75 were accepted. This cutoff is based on an unpublished study, where we observed a negative correlation between the percentage of incorrect variant calls and increasing read depth. The curve plateaued at a read depth of 75. The variant type was changed to heterozygote variant calls if the allele balance (reference allele / reference allele + alternative allele) was between 0.32 and 0.68. All DNA analyses were carried out using *RStudio version 1*.*4*.*1717* [27] with *R version 4*.*1*.*1* [22] with the use of packages, *GenomicRanges*, *data*.*table*, and *tidyverse*.

## Results

WTS of RNA extracted from paired fresh, frozen, and FFPE cardiac tissue was performed to assess the effect of storage conditions on the gene expression profiles. Protein-coding RNAs, mtRNAs, and lncRNAs were investigated separately to assess RNA type-specific effects of storage conditions.

### The quality of extracted RNA and yield of sequencing

The RIN and DV200 were calculated to evaluate the quality of RNA extracted from fresh, frozen, and FFPE cardiac tissue. RNA extracted from FFPE tissue was of markedly lower quality (median RIN = 2.5, median DV200 = 48%) compared to RNA extracted from both fresh and frozen tissue (median RIN = 8.1, median DV200 = 97%; median RIN = 8.0, median DV200 = 96%, respectively) (S1A and S1B Fig, S1 Table).

The RNA sequencing generated a median of ~ 65 million reads per sequencing library (range: 42–204 million reads). One of three replicates from either the fresh, frozen, or FFPE tissue from four patients was omitted from further analyses due to failed sequencing, suspected DNA contamination, or gene expression profiles suggesting contamination with adipose tissue. There was a high correlation between gene expression patterns in replicate investigations (average ρ: fresh = 0.89, frozen = 0.87, FFPE = 0.83) (S2 Fig), and the replicate gene expression values were summed in subsequent analyses.

### Alignment statistics and relative abundance of RNA subtypes

**Fig 2:**
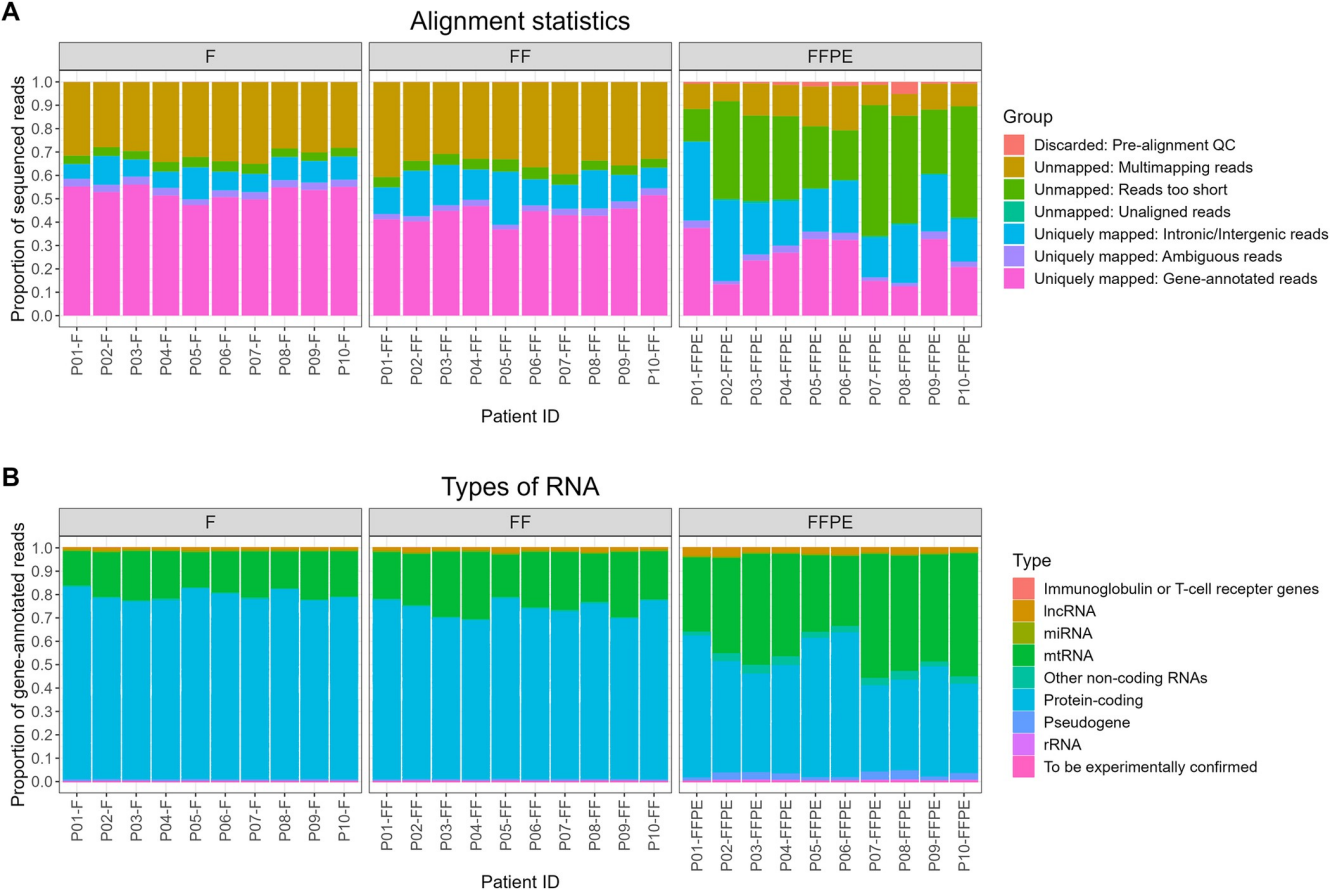
Alignment statistics and RNA subtypes from fresh, frozen, and formalin-fixed, paraffin-embedded (FFPE) cardiac tissue. **(A)** Stacked bar plots of the proportions of unmapped and uniquely mapped reads per patient. **(B)** Stacked bar plots of the proportions of RNA subtypes. Abbreviations: F = Fresh tissue, FF = Frozen tissue, lncRNA = Long non-coding RNA, miRNA = MicroRNA, mtRNA = Mitochondrial RNA, rRNA = Ribosomal RNA, QC = Quality control.

Alignment statistics were investigated to assess the proportion of successfully aligned reads. Alignment statistics were similar for fresh and frozen tissues and were consistent among all patients (Fig 2A). In general, both fresh and frozen tissues yielded large proportions of reads aligning to regions in which active transcription is expected (hereafter referred to as gene-annotated reads) (average of 53% and 44% gene-annotated reads, respectively). Both fresh and frozen tissue yielded relatively large proportions of multi-mapping reads (average of 31% and 35% multi-mapping reads, respectively), which is expected when performing short-read sequencing. FFPE tissues had large proportions of reads that were too short to align or aligned to intronic/intergenic regions (Fig 2A). This was, however, expected due to the low quality of RNA extracted from FFPE tissue, and is most likely the result of formalin-induced RNA degradation. In addition, inter-individual differences in alignment statistics were observed in the FFPE tissues (Fig 2A). The inter-individual differences in alignment statistics for FFPE samples did not seem to result from fixation- or storage times (S3 Fig). Only uniquely mapping gene-annotated reads were used in subsequent analyses. High RNA quality scores (RIN and DV200) were associated with increased proportions of gene-annotated reads (S4 Fig). Slightly lower GC content was observed in aligned reads from FFPE tissue than fresh and frozen tissues (S5 Fig).

Most gene-annotated reads originated from protein-coding genes (fresh: 76%, frozen 73%, and FFPE: 48%) (Fig 2B). However, higher proportions of RNAs aligning to mtRNA and lncRNA genes were observed in FFPE tissue compared to fresh and frozen tissue. The RNA extraction protocols used in this study did not include microRNAs and other short RNA types, whereby they are absent from the sequencing data.

### Gene expression profiles of the protein-coding, mitochondrial, and long non-coding RNA subsets

**Fig 3:**
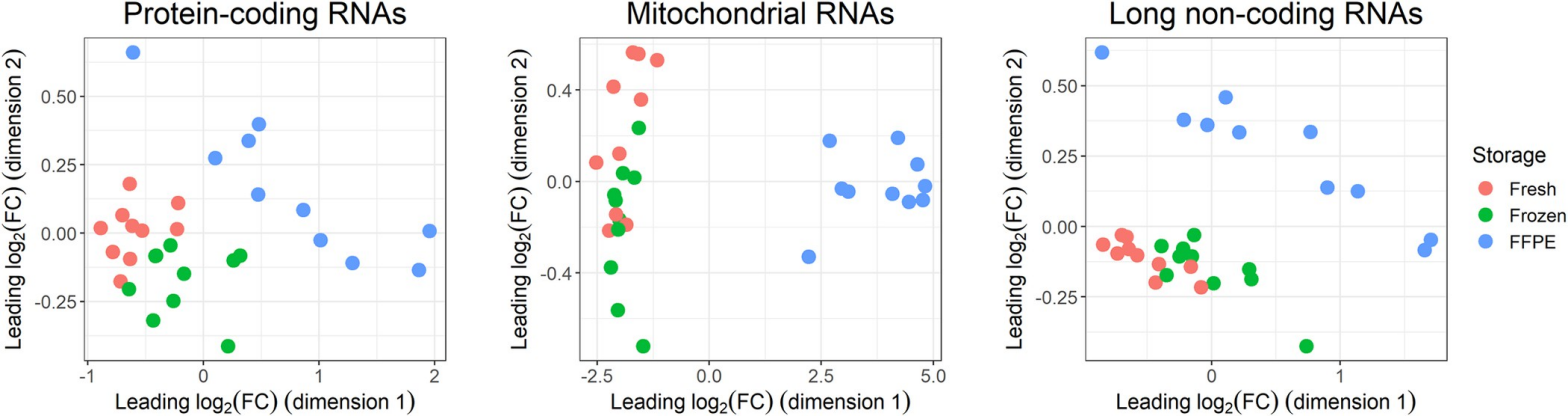
Multidimensional scaling plots of gene expression profiles of the protein-coding, mitochondrial, and long non-coding RNA subsets. Colours correspond to storage condition (red = fresh tissue, green = frozen tissue, blue = FFPE tissue). Abbreviations: FC = Fold-change, FFPE = Formalin-fixed, paraffin-embedded.

MDS plots were generated to assess the effect of storage conditions on the protein-coding RNA, mtRNA, and lncRNA subsets. For all three RNA subsets, a clear separation of storage conditions was observed (Fig 3). No such separation of inter-individual variation was observed (S6 Fig). Hence, in this dataset, the overall variation induced by storage conditions seems larger than the biological differences among individuals.

**Fig 4:**
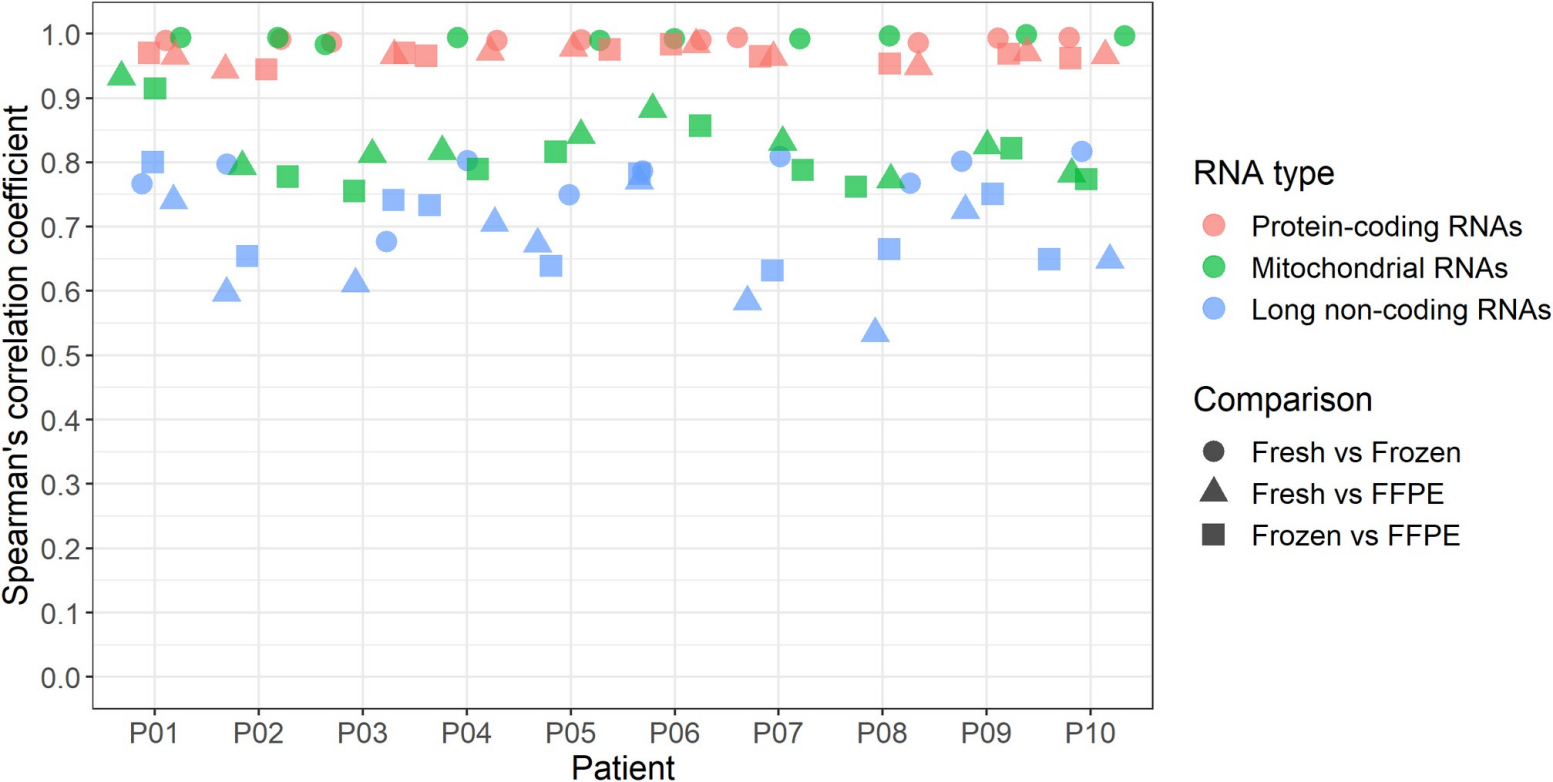
Spearman’s correlation coefficients of intra-individual gene expression levels from fresh, frozen, and formalin-fixed, paraffin-embedded (FFPE) tissue. Correlations were calculated for all 10 patients based on the protein-coding, mitochondrial, and long non-coding RNAs when comparing fresh, frozen, and FFPE tissues.

Spearman’s correlation analyses were performed to assess the intra-individual ranked gene (ranked by log2(CPM)) concordance between fresh, frozen, and FFPE tissues. A very high correlation between protein-coding transcripts was observed when comparing all three storage conditions (ρ > 0.94) (Fig 4, S7 Fig). For the mitochondrial subset, a very high correlation was observed when comparing gene expression profiles from fresh and frozen tissues (ρ > 0.98) (Fig 4, S7 Fig). However, a subset of mitochondrial transcripts seemed falsely overexpressed in FFPE tissue compared to fresh and frozen tissues (S7 Fig). The Spearman’s correlation coefficients between gene expression levels of lncRNAs in the three different storage conditions were lower than protein-coding and mitochondrial RNAs (ρ > 0.53) (Fig 4, S7 Fig). For both the protein-coding and lncRNA subsets, most of the variation between storage conditions was observed in genes with low expression levels (S7 Fig).

We investigated if the effect of storage conditions could be reduced by filtering out genes with low expression levels. Thresholds of both log_2_(CPM) > 0 and log_2_(CPM) > 5 were investigated. MDS-plots of the filtered data, nevertheless, showed clustering based on storage conditions. However, a reduction of the overall variation caused by storage conditions was observed using both filtering thresholds (S8–S10 Figs). In addition, plotting dimensions 2 and 3 in the MDS-plot of the protein-coding subset revealed a separation of patients, whereby inter-individual differences were now observed (S11 Fig).

### Concordance between RNA and DNA variants

**Fig 5:**
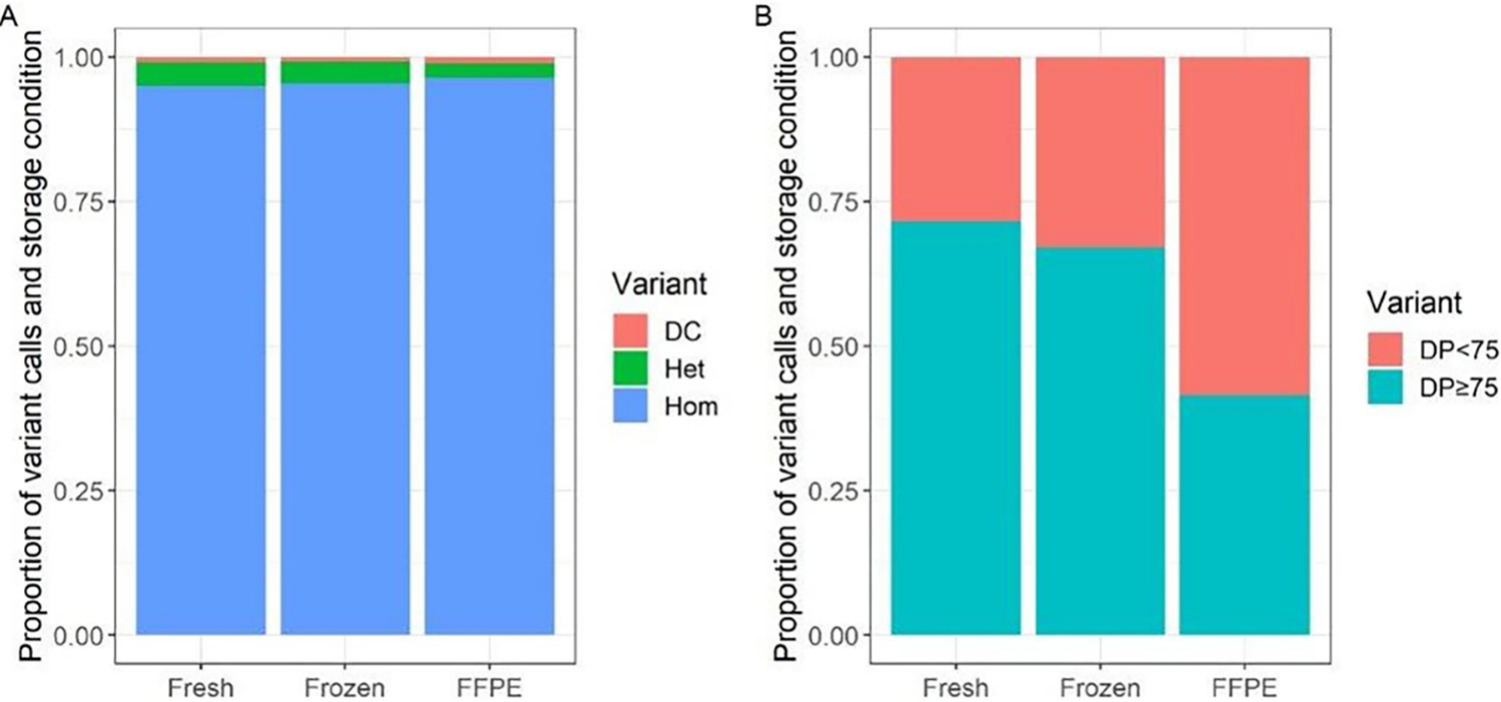
Concordance between DNA and RNA variants among different storage conditions. **(A)** Proportion of concordant (Het = heterozygous, Hom = homozygous) and discordant (DC) variant calls in loci with read depth (DP) ≥ 75. **(B)** Proportion of detected variants with read depths too low (DP<75) for reliable variant calls. Abbreviations: FFPE = Formalin-fixed, paraffin-embedded.

We investigated RNA sequencing variants from the WTS data in exon regions of 31 cardiac genes and compared them with DNA variants identified with the OMNI5-4 single nucleotide polymorphism (SNP) array. The genes were reported as cardiac disease-related genes in the ACMG SF v3.0 list [25] or genes implicated in atrial fibrillation [26]. A complete list of the genes is found in S2 Table. Overall, we found a high concordance between DNA and RNA variants across all storage conditions (Fig 5A). We did not find ASE in any of the investigated cardiac genes among the different storage conditions. However, we found the FFPE tissue to have a statistically significantly (p-value < 0.05) higher number (odds ratio = 1.65) of discordant variant calls compared with fresh and frozen tissues. In addition, we found larger proportions of variants with insufficient read depths (< 75) for reliable variant calls in FFPE compared to fresh and frozen tissues (Fig 5B). We did not observe discordant variant calls for any of the genes associated with atrial fibrillation including *SNC5A*, *KCNQ1*, *KCNH2*, *TBX5*, *GJA5*, *MYL4*, *TTN*, and *KCNA5*, whereas we found a few discordant variant calls in some of the cardiac genes on the ACMG list, including *DSG2*, *DSP*, *LMNA*, *MYH7*, and *TPM1*. However, the patterns of discordant calls within each gene did not support ASE (S12 Fig).

## Discussion

Tissues are routinely fixed in formalin and embedded in paraffin to ensure easy long-term storage at room temperature. Hence, repositories worldwide in forensic and pathology departments represent a valuable tissue source for retrospective studies. However, as formalin-fixation degrades nucleic acids, the gold standard for genetic and gene expression analysis has been to use fresh or frozen tissue. We, therefore, investigated the utility of using both frozen and FFPE tissue for gene expression analysis by comparison with paired fresh tissue.

Total RNA was successfully extracted from fresh, frozen, and FFPE cardiac tissues. However, the quality, assessed using the RIN and DV200 scores, varied between storage conditions. High-quality RNA was extracted from both fresh and frozen tissues, whereas quality scores for RNA extracted from FFPE tissues indicated significant degradation.

The alignment of RNA sequencing data from FFPE tissues yielded larger proportions of unmapped reads than fresh and frozen tissues. This is most likely due to formalin-induced degradation of RNA. In addition, larger proportions of off-target intergenic and intronic reads were observed in FFPE tissue compared to fresh and frozen tissues. This pattern has been observed in several previous studies, and it has been suggested to be the result of formalin-induced cross-linking and degradation of mature cytoplasmic transcripts [11, 12, 28, 29]. In line with other studies, we observed a slightly lower GC content in FFPE tissues than fresh and frozen tissues [10, 29].

The degradation of cytoplasmic transcripts may also explain the differences in the proportions of protein-coding RNAs, mtRNAs, and lncRNAs. The proportions of mtRNAs and lncRNAs relative to the proportion of protein-coding RNAs were higher in FFPE tissue than in fresh and frozen tissues. One could speculate that cytoplasmic protein-coding transcripts may be more susceptible to formalin-induced degradation compared to mitochondrial transcripts that may be protected by the mitochondrial membrane. In a previous study, we observed an association between the level of post-mortem degradation of cardiac tissues and the proportion of mitochondrial transcripts in WTS data, indicating a non-uniform degradation of RNA types within the cells [30]. However, this hypothesis needs further investigation.

When assessing the global gene expression profiles of protein-coding RNAs, mtRNAs, and lncRNAs, the largest source of variation was the tissue storage condition. The variation induced by storage condition was, in this study, larger than the inter-individual differences. Most of the variation was observed in transcripts with low expression. We filtered the RNA sequencing dataset based on the arbitrary thresholds log_2_(CPM)>0 and log_2_(CPM)>5. This reduced the overall variation caused by storage conditions but did not eliminate it completely. In addition, when filtering the data, large proportions of the data were removed as many genes had low expression levels. We do not have enough evidence to determine an appropriate threshold for filtering. However, we call for caution when studying genes with low expression.

For protein-coding transcripts, we observed a very high correlation between global gene expression profiles when comparing paired fresh, frozen, and FFPE tissues. However, mitochondrial and long non-coding transcripts’ gene expression profiles seemed to be more affected by storage conditions. Nevertheless, due to the overall high concordance in gene expression patterns, we find that fresh, frozen, and FFPE tissues can be used for gene expression analyses. However, we recommend not comparing gene expression in tissues with different storage conditions to minimize the bias induced by storage conditions. Due to the small sample size in this study, we decided not to report differences at the gene level as such findings are associated with a high false discovery rate. Hence, a larger number of samples must be studied to investigate if specific genes have increased technical variability caused by storage conditions.

We found a statistically significantly (p < 0.05) higher number of DNA-RNA discordant variant calls in the FFPE tissue compared with those found in the fresh and frozen tissue. Discordant variant calls may be an indication of ASE if heterozygous DNA variants show only one allele in the RNA. However, the higher number observed in this study were artifacts and highlight a potential pitfall in ASE analysis using FFPE tissues. We did not observe discordant variant calls in the fresh tissue for the investigated genes associated with atrial fibrillation. Our results show that both alleles of these genes were expressed (biallelic gene expression). However, we saw discordant calls in other cardiac genes. The patterns of discordant calls within the various genes did not support ASE of any of the investigated genes.

We observed a larger proportion of loci with read depths insufficient for reliable variant calls (< 75) in FFPE tissue compared to fresh and frozen tissue. This may be due to the degradation of RNA molecules in FFPE tissue that results in a reduction of successfully aligned reads. This could be accounted for by increasing the sequencing depth (the total number of reads per sample) when working with FFPE tissues.

We performed WTS on cardiac tissues to investigate the utility of fresh, frozen, and FFPE tissues for gene expression analysis in cardiac diseases. The lower quality of RNA from FFPE than frozen tissue has been seen in other tissues [10, 12, 31], whereas the comparison of RNA from fresh, frozen, and FFPE is new. We hypothesise that the results from this study apply to other tissues. However, future studies need to confirm this.

The paraffin must be removed from FFPE tissues before extracting the RNA. It is, therefore, a limitation of the study that the protocol for RNA extraction from FFPE tissue differed slightly from that of fresh and frozen tissue. We can, hence, not conclude that the differences observed are results of fixation or freezing/thawing. The variation in RNA quality and sequencing results may be a result of both the storage condition and RNA extraction method.

Additionally, the time of storage in this study was short compared to the repositories of tissues available worldwide. Storage duration has been shown to affect the quality of both DNA and RNA, whereby differences in gene expression profiles may be more pronounced with long-term storage [32, 33]. However, further investigations are needed to identify the individual effects of the storage method, nucleic acids extraction method, and long-term storage on gene expression profiles.

## Conclusion

Overall, we found that fresh, frozen, and FFPE tissues can be used to generate reliable gene expression profiles. However, proper quality control is essential. We recommend refraining from comparing gene expression in tissues with different storage conditions. In addition, it is essential to be critical when reporting differences in gene expression of genes with low expression, as this may be due to technical variability. Also, FFPE gave more discordant DNA-RNA variant calls compared to fresh and fresh frozen tissue. Being aware of the limitations and pitfalls of working with FFPE tissue, we are confident that the immense resources of frozen and FFPE tissues can be utilized to assess gene expression in many human diseases.

## Supporting information

S1 Fig Quality scores of RNA extracted from fresh, frozen, and formalin-fixed, paraffin-embedded (FFPE) cardiac tissue. **(A)** Box plots of the RNA integrity number (RIN) of RNA extracted from fresh, frozen, and FFPE tissue (n = 78). **(B)** Box plots of the percentage of RNA fragments > 200 nucleotides (DV200) of RNA extracted from fresh, frozen, and FFPE tissue (n = 84). Whiskers represent data points within the 25^th^ percentile– 1.5 · interquartile range (IQR) and the 75^th^ percentile + 1.5 · IQR. Dots represent data points outside these intervals.
(TIF)

S2 Fig Spearman’s correlation analysis of replicate RNA sequencing investigations in fresh, frozen, and FFPE tissues. Diagonal plots show sample ID, the lower panel shows scatter plots of the paired comparison between gene expression in terms of the log_2_(counts-per-million), and the upper panel states the Spearman’s correlation coefficient (rho) and p-value (p).
(PDF)

S3 Fig Scatter plots of the proportions of gene-annotated reads and fixation + storage times for FFPE tissues. (TIF)

S4 Fig Scatter plots of the proportions of gene-annotated reads and RNA quality scores. (TIF)

S5 Fig GC content of mapped sequencing reads. (TIF)

S6 Fig Multidimensional scaling (MDS) plots of gene expression profiles of the protein-coding, mitochondrial, and long non-coding RNA subsets. Colours correspond to patient ID. Abbreviations: FC = Fold-change.
(TIF)

S7 Fig Spearman’s correlation of protein-coding RNAs, mitochondrial RNAs, and long non-coding RNAs in fresh, frozen, and FFPE tissues for all patients. Diagonal plots show sample ID, the lower panel shows scatter plots of the paired comparison between gene expression in terms of the log_2_(counts-per-million), and the upper panel states the Spearman’s correlation coefficient (rho) and p-value (p).
(PDF)

S8 Fig Multidimensional scaling (MDS) plots of gene expression profiles of the filtered protein-coding RNA subset. Abbreviations: CPM = Counts-per-million, FC = Fold-change, FFPE = Formalin-fixed, paraffin-embedded. (MDS-plots of unfiltered data is shown in Fig 3 and S6 Fig (n = 19,461 genes)).
(PDF)

S9 Fig Multidimensional scaling (MDS) plots of gene expression profiles of the filtered mitochondrial RNA subset. Abbreviations: CPM = Counts-per-million, FC = Fold-change, FFPE = Formalin-fixed, paraffin-embedded. (MDS-plots of unfiltered data is shown in Fig 3 and S6 Fig (n = 37 genes)).
(PDF)

S10 Fig Multidimensional scaling (MDS) plots of gene expression profiles of the filtered long non-coding RNA subset. Abbreviations: CPM = Counts-per-million, FC = Fold-change, FFPE = Formalin-fixed, paraffin-embedded. (MDS-plots of unfiltered data is shown in Fig 3 and S6 Fig (n = 16,598 genes)).
(PDF)

S11 Fig Multidimensional scaling (MDS) plots (dimensions 2 and 3) of gene expression profiles of the unfiltered and filtered protein-coding RNA subset. Abbreviations: CPM = Counts-per-million, FC = Fold-change, FFPE = Formalin-fixed, paraffin-embedded.
(PDF)

S12 Fig Concordance between DNA and RNA variants per gene among storage conditions. Numbers within the bar plots display the number of observations for each variant type. Abbreviations: FFPE = Formalin-fixed, paraffin-embedded, DC: Discordant calls, Het = Heterozygous, Hom = Homozygous.
(TIF)

S1 Table RIN and DV200 scores of RNA extracted from fresh, frozen, and FFPE tissues. (XLSX)

S2 Table Gene regions included in the assessment of the concordance between RNA and DNA variants. (XLSX)
