# Supplementary figures and images for: Comparison of whole transcriptome sequencing of fresh, frozen, and formalin-fixed, paraffin-embedded cardiac tissue
Source: PLoS One. 2023 Mar 29;18(3):e0283159. doi: 10.1371/journal.pone.0283159 (PMC10058139; doi:10.1371/journal.pone.0283159)

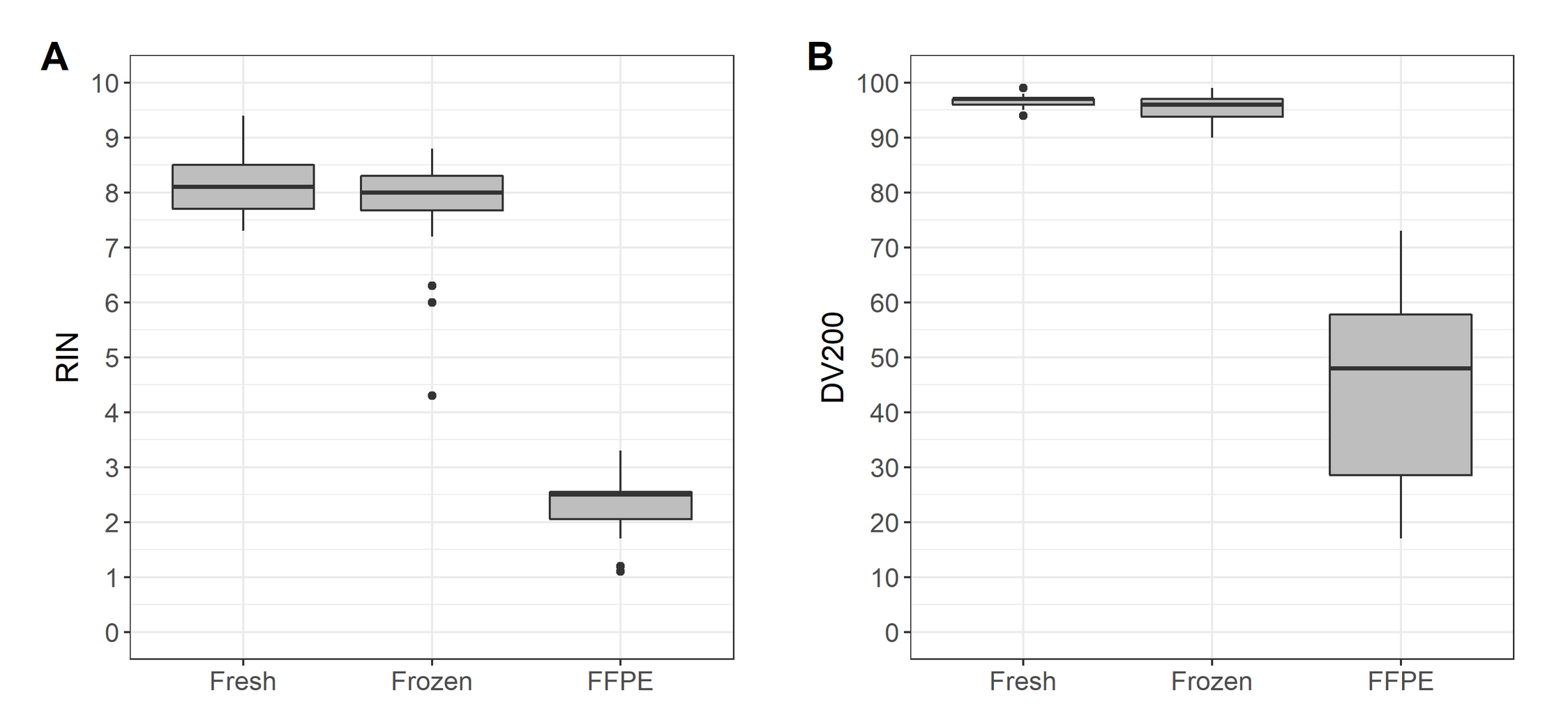

Supplement: S1 Fig — (A) Box plots of the RNA integrity number (RIN) of RNA extracted from fresh, frozen, and FFPE tissue (n = 78). (B) Box plots of the percentage of RNA fragments > 200 nucleotides (DV200) of RNA extracted from fresh, frozen, and FFPE tissue (n = 84). Whiskers represent data points within the 25th percentile– 1.5 · interquartile range (IQR) and the 75th percentile + 1.5 · IQR. Dots represent data points outside these intervals. (TIF) [file pone.0283159.s001.tif]

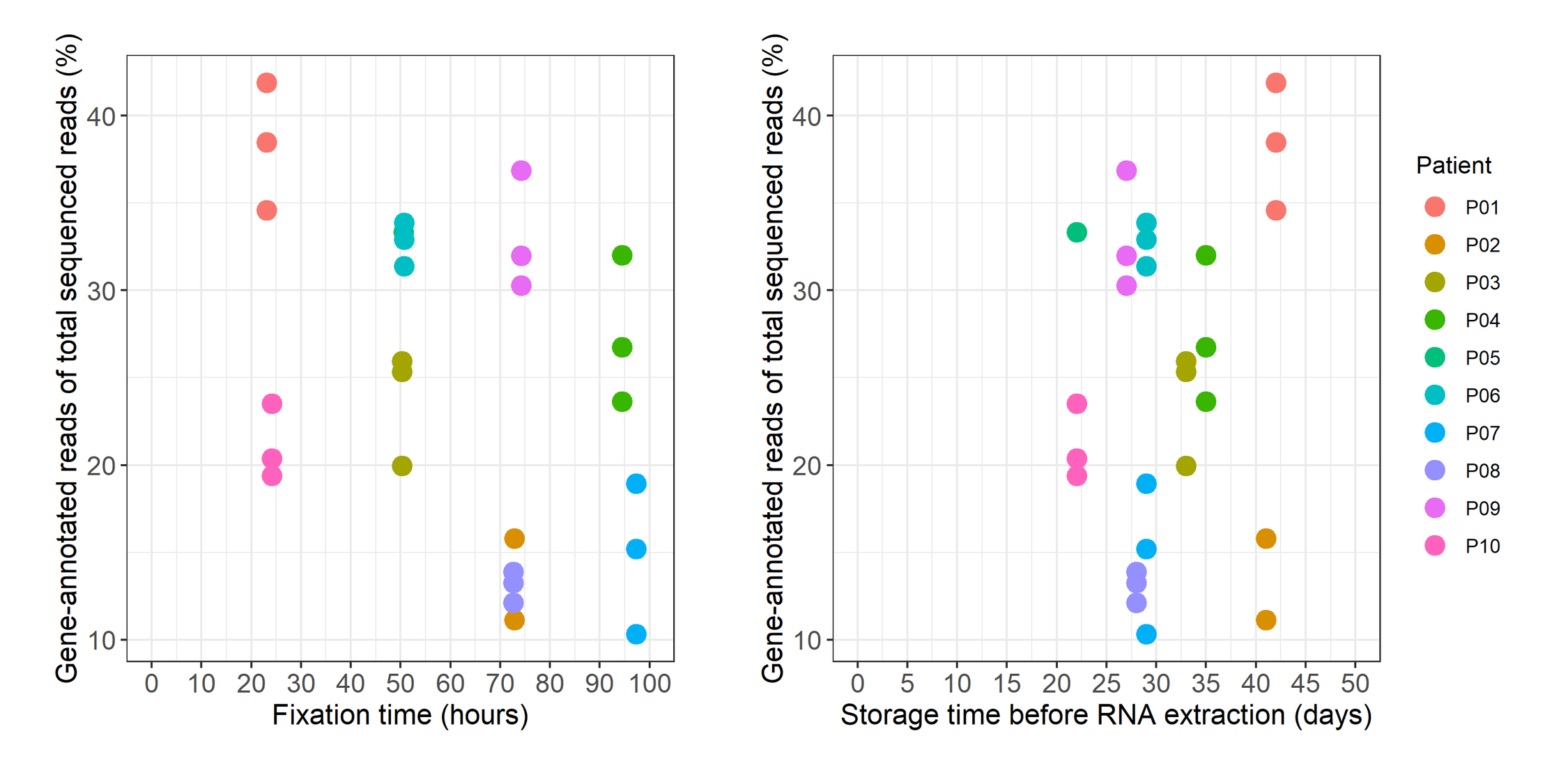

Supplement: S3 Fig — (TIF) [file pone.0283159.s003.tif]

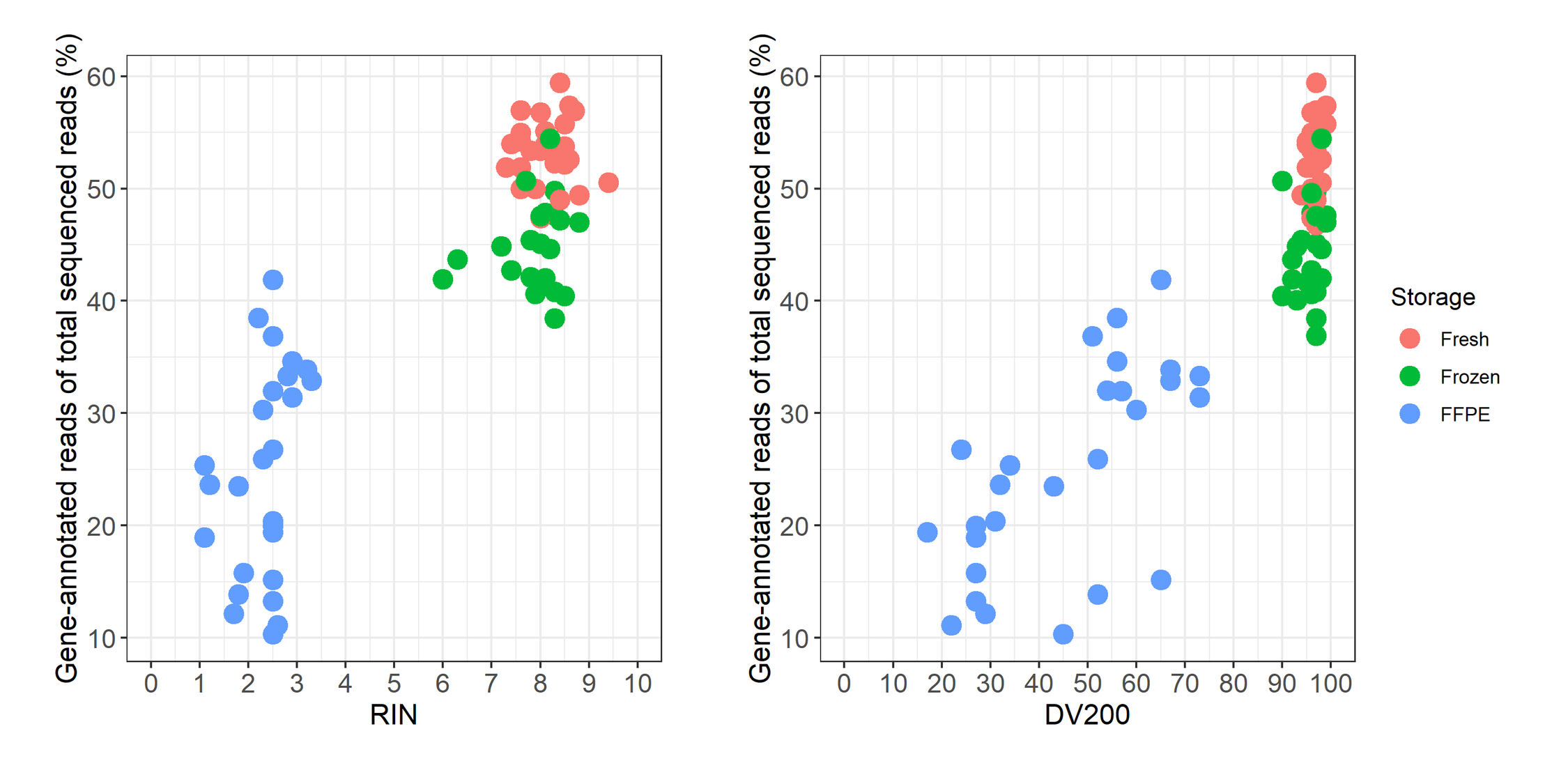

Supplement: S4 Fig — (TIF) [file pone.0283159.s004.tif]

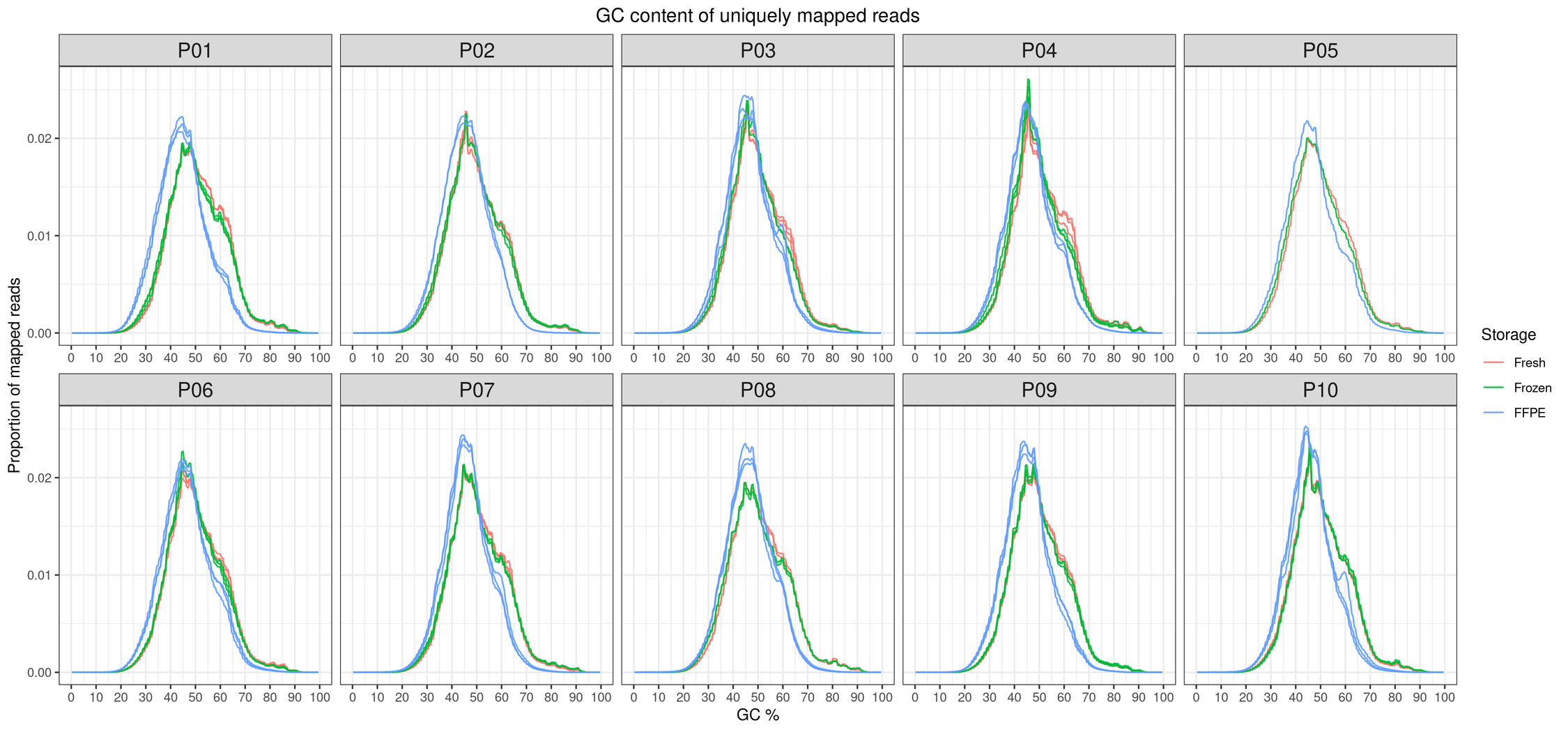

Supplement: S5 Fig — (TIF) [file pone.0283159.s005.tif]

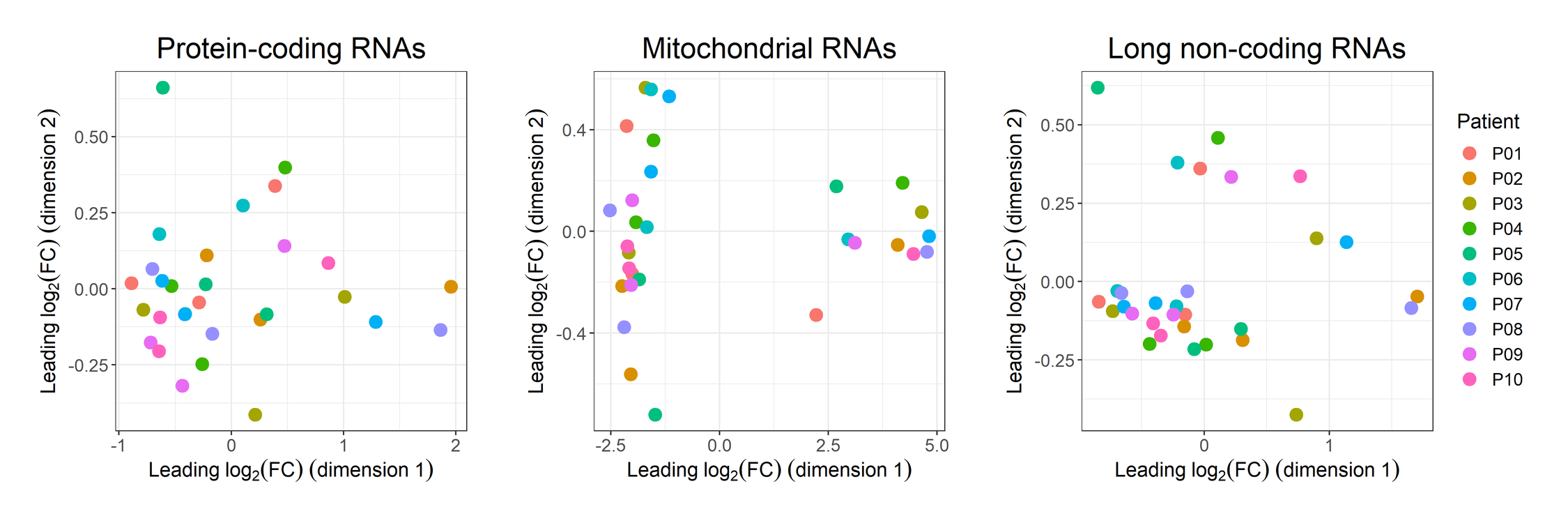

Supplement: S6 Fig — Colours correspond to patient ID. Abbreviations: FC = Fold-change. (TIF) [file pone.0283159.s006.tif]

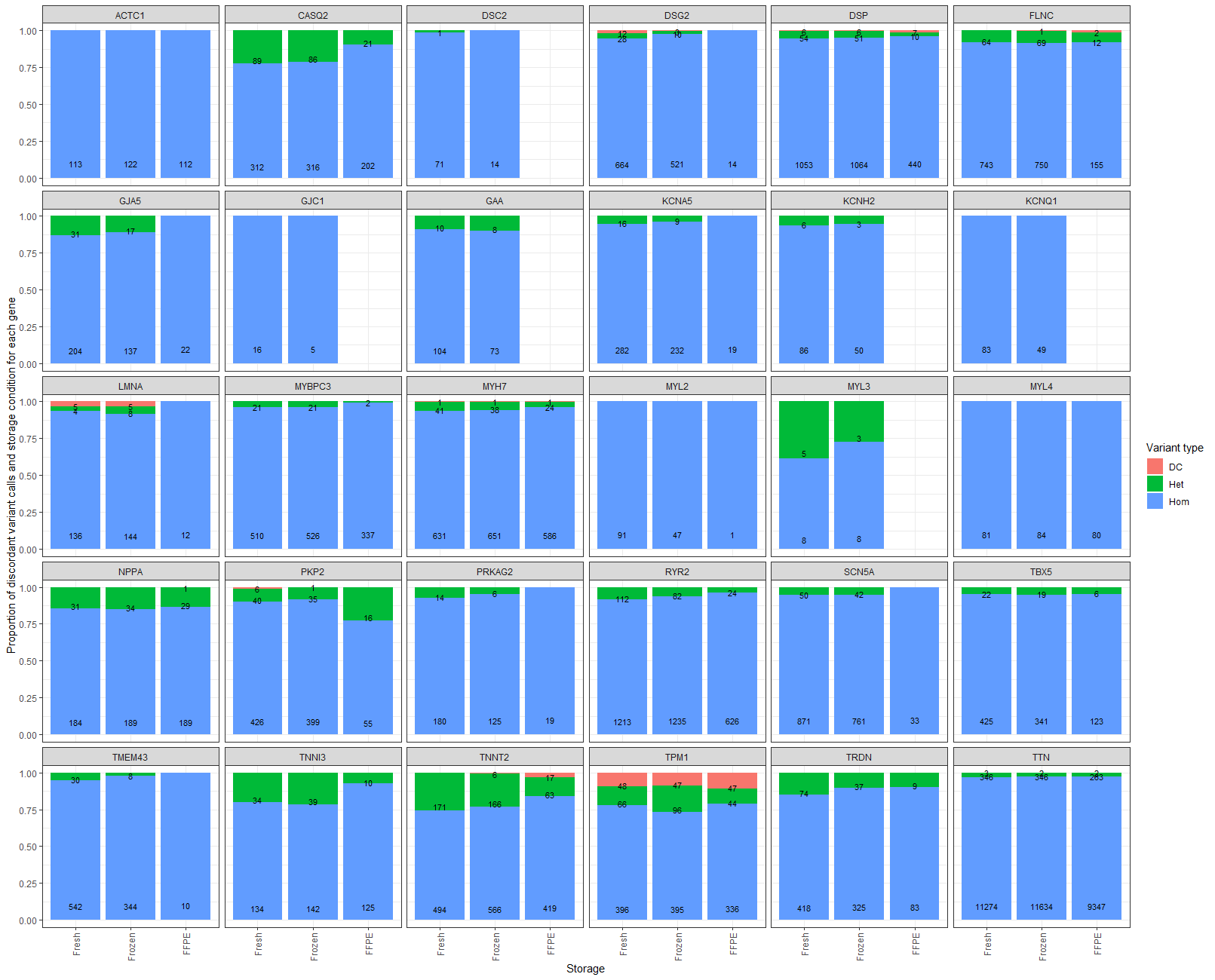

Supplement: S12 Fig — Numbers within the bar plots display the number of observations for each variant type. Abbreviations: FFPE = Formalin-fixed, paraffin-embedded, DC: Discordant calls, Het = Heterozygous, Hom = Homozygous. (TIF) [file pone.0283159.s012.tif]
