# Supplementary material for: Comparison of whole transcriptome sequencing of fresh, frozen, and formalin-fixed, paraffin-embedded cardiac tissue
Source: PLoS One. 2023 Mar 29;18(3):e0283159. doi: 10.1371/journal.pone.0283159 (PMC10058139; doi:10.1371/journal.pone.0283159)
Supplement: S2 Fig — Diagonal plots show sample ID, the lower panel shows scatter plots of the paired comparison between gene expression in terms of the log2(counts-per-million), and the upper panel states the Spearman’s correlation coefficient (rho) and p-value (p). (PDF) [file pone.0283159.s002.pdf]

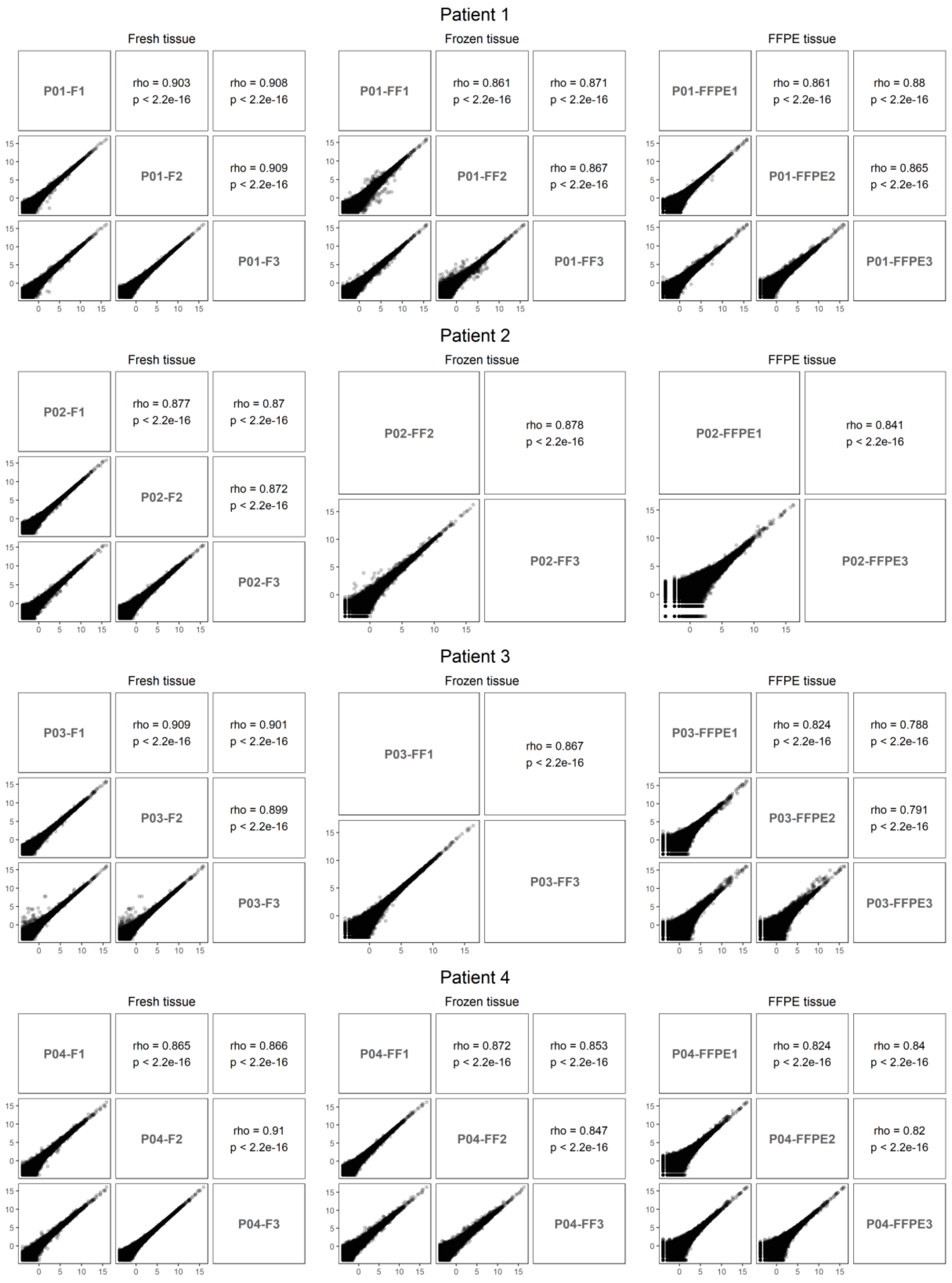

S2 Fig: Spearman's correlation analysis of replicate RNA sequencing investigations in fresh, frozen, and FFPE tissues. Diagonal plots show sample ID, the lower panel shows scatter plots of the paired comparison between gene expression in terms of the $\log_2$(counts-per-million), and the upper panel states the Spearman's correlation coefficient (rho) and p-value (p).

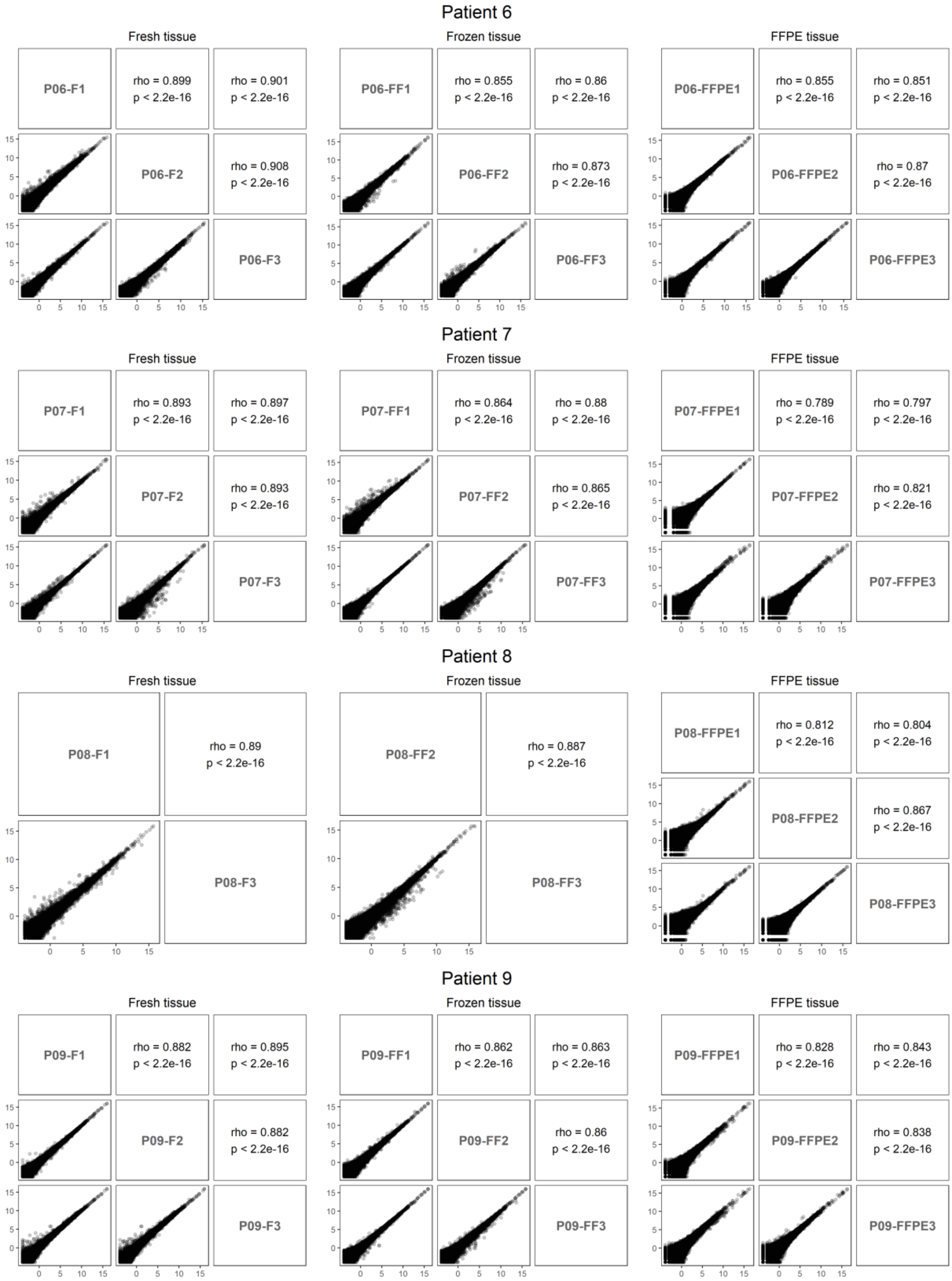

S2 Fig: Spearman's correlation analysis of replicate RNA sequencing investigations in fresh, frozen, and FFPE tissues - continued.

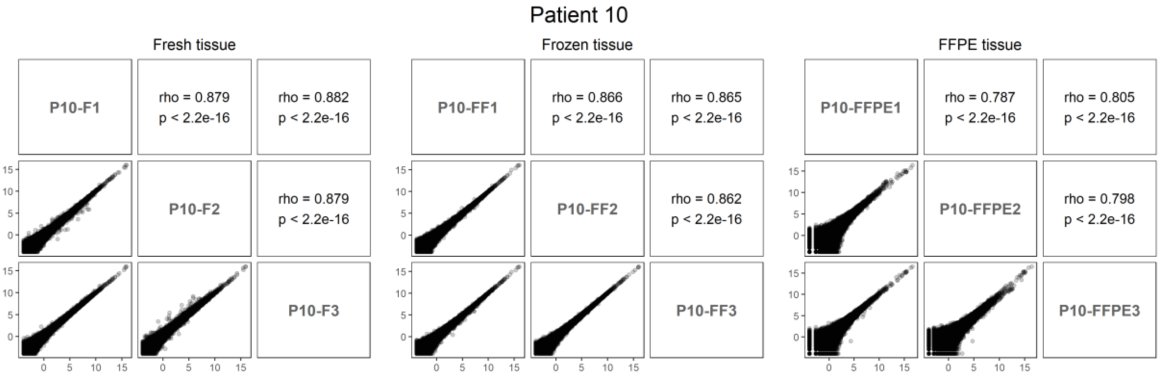

S2 Fig: Spearman's correlation analysis of replicate RNA sequencing investigations in fresh, frozen, and FFPE tissues - continued.
