# Supplementary material for: Comparison of whole transcriptome sequencing of fresh, frozen, and formalin-fixed, paraffin-embedded cardiac tissue
Source: PLoS One. 2023 Mar 29;18(3):e0283159. doi: 10.1371/journal.pone.0283159 (PMC10058139; doi:10.1371/journal.pone.0283159)
Supplement: S7 Fig — Diagonal plots show sample ID, the lower panel shows scatter plots of the paired comparison between gene expression in terms of the log2(counts-per-million), and the upper panel states the Spearman’s correlation coefficient (rho) and p-value (p). (PDF) [file pone.0283159.s007.pdf]

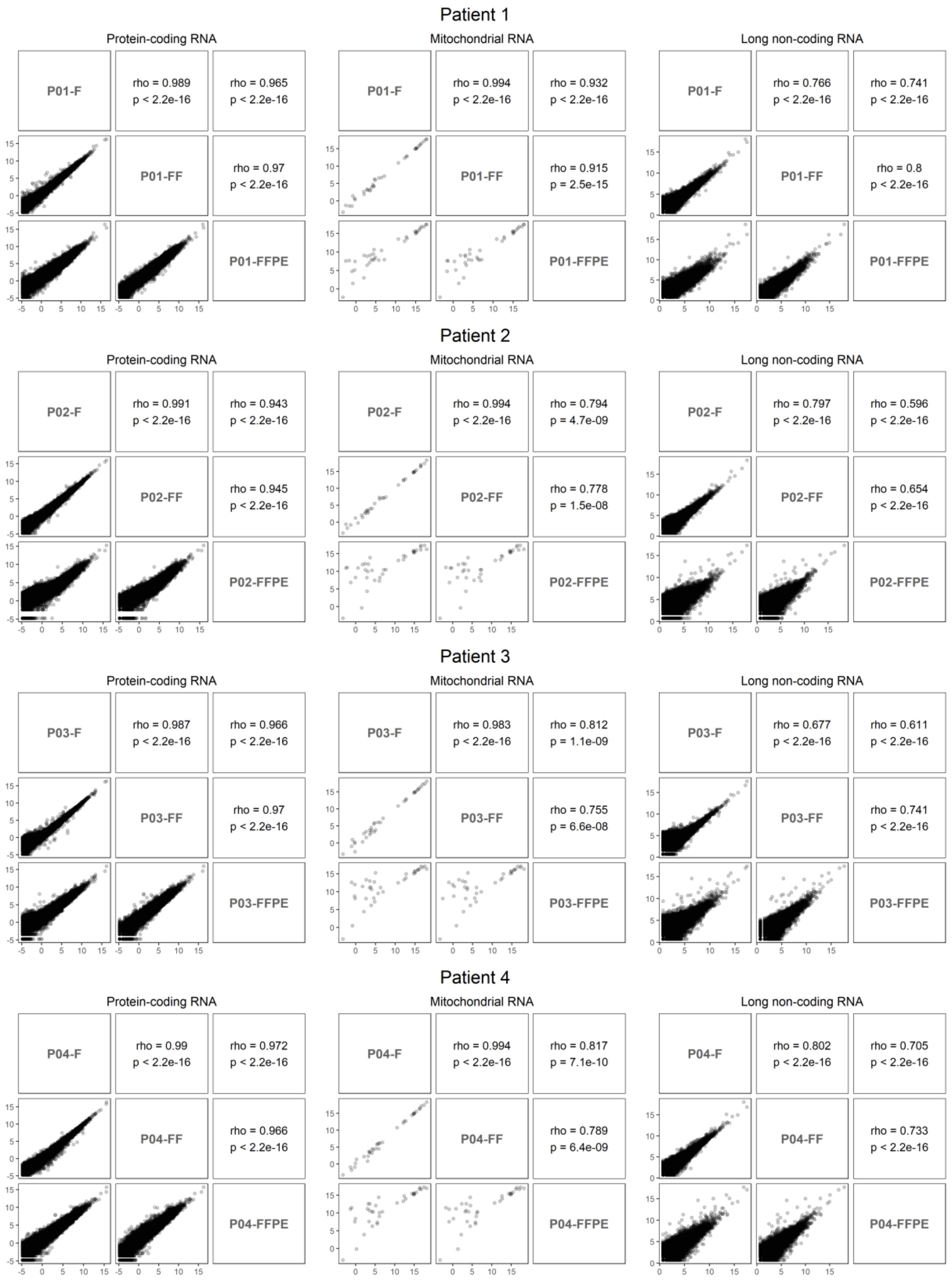


**S7 Fig: Spearman's correlation of protein-coding RNAs, mitochondrial RNAs, and long non-coding RNAs in fresh, frozen, and FFPE tissues for all patients.**

Diagonal plots show sample ID, the lower panel shows scatter plots of the paired comparison between gene expression in terms of the $\log_2$(counts-per-million), and the upper panel states the Spearman's correlation coefficient (rho) and p-value (p).

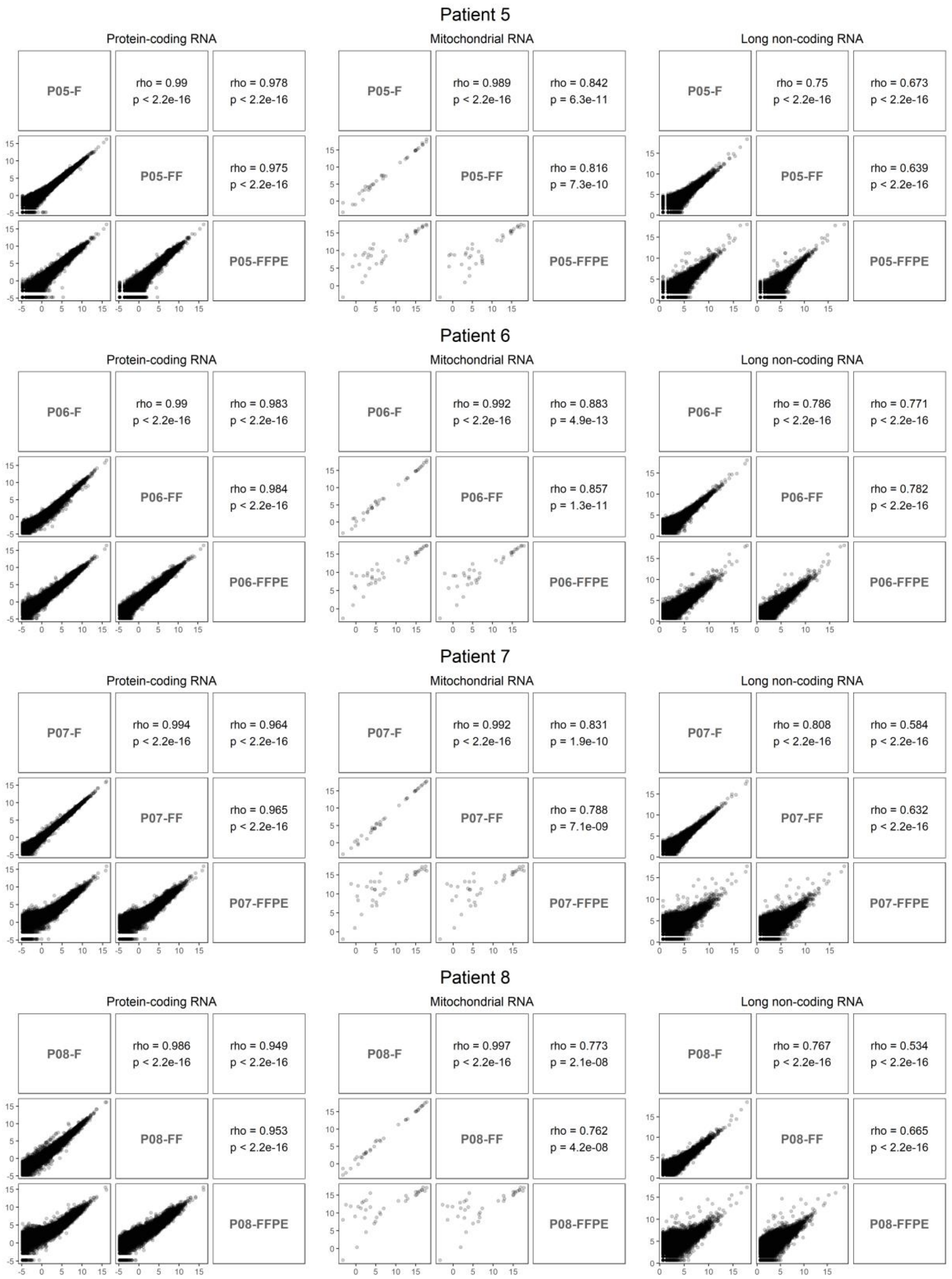


**S7 Fig: Spearman's correlation of protein-coding RNAs, mitochondrial RNAs, and long non-coding RNAs in fresh, frozen, and FFPE tissues for all patients - continued.**

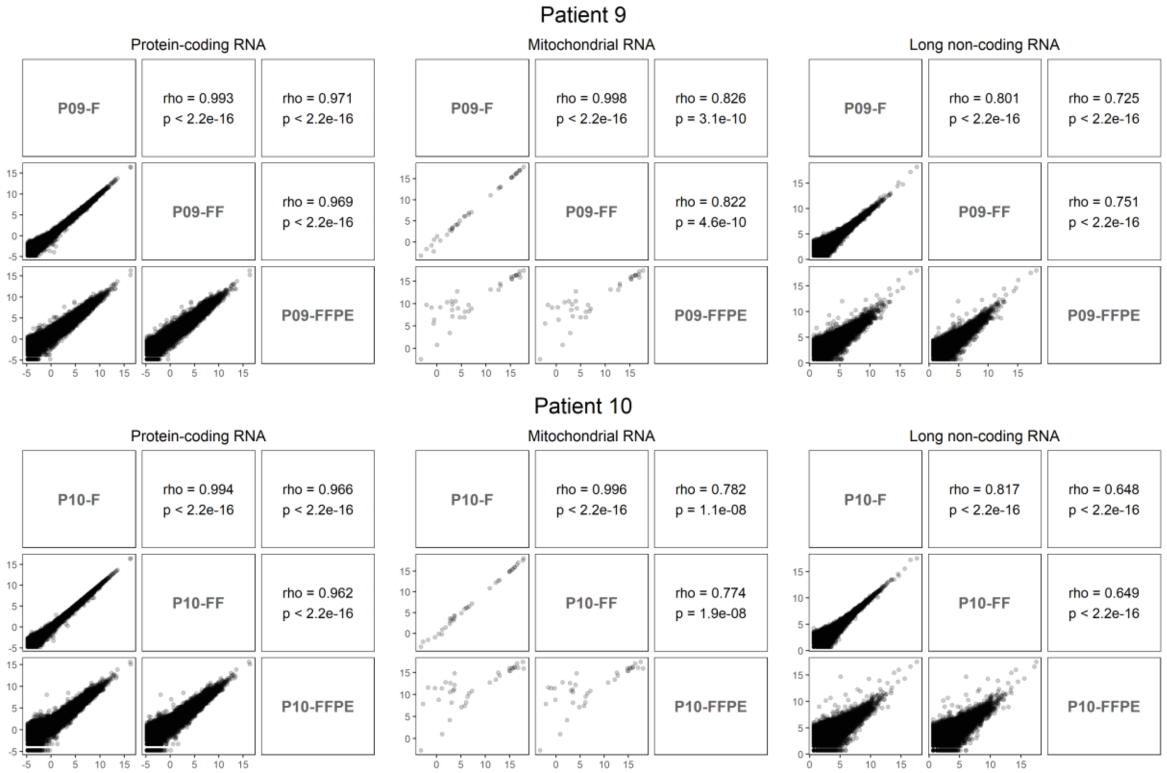


**S7 Fig: Spearman's correlation of protein-coding RNAs, mitochondrial RNAs, and long non-coding RNAs in fresh, frozen, and FFPE tissues for all patients - continued.**
