# Supplementary material for: Comparison of whole transcriptome sequencing of fresh, frozen, and formalin-fixed, paraffin-embedded cardiac tissue
Source: PLoS One. 2023 Mar 29;18(3):e0283159. doi: 10.1371/journal.pone.0283159 (PMC10058139; doi:10.1371/journal.pone.0283159)
Supplement: S9 Fig — Abbreviations: CPM = Counts-per-million, FC = Fold-change, FFPE = Formalin-fixed, paraffin-embedded. (MDS-plots of unfiltered data is shown in Fig 3 and S6 Fig (n = 37 genes)). (PDF) [file pone.0283159.s009.pdf]

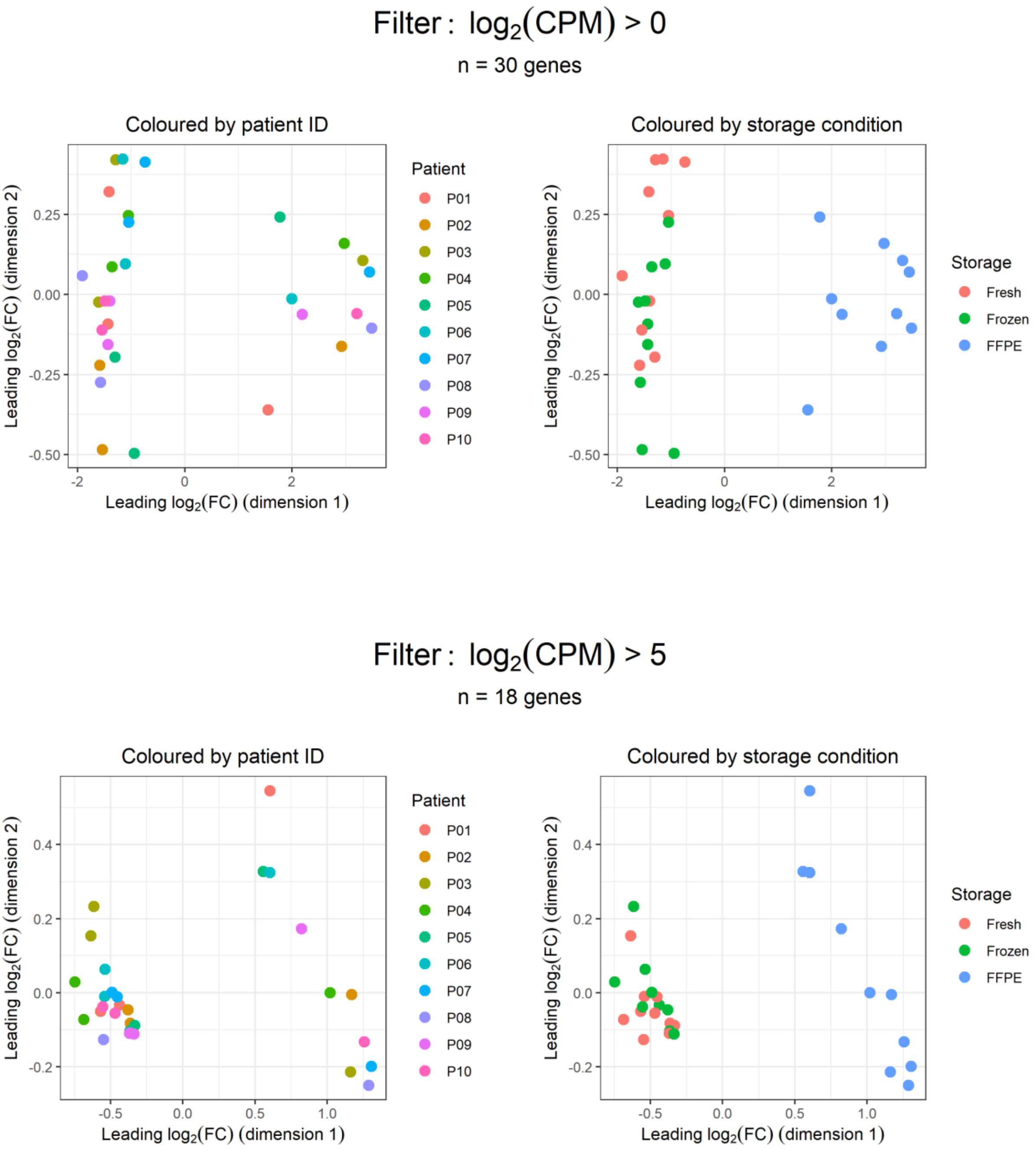

S9 Fig: Multidimensional scaling (MDS) plots of gene expression profiles of the filtered mitochondrial RNA subset. Abbreviations: CPM = Counts-per-million, FC = Fold-change, FFPE = Formalin-fixed, paraffin-embedded. (MDS-plots of unfiltered data is shown in Figs 3 and S6 (n = 37 genes)).
