# Supplementary material for: Comparison of whole transcriptome sequencing of fresh, frozen, and formalin-fixed, paraffin-embedded cardiac tissue
Source: PLoS One. 2023 Mar 29;18(3):e0283159. doi: 10.1371/journal.pone.0283159 (PMC10058139; doi:10.1371/journal.pone.0283159)
Supplement: S11 Fig — Abbreviations: CPM = Counts-per-million, FC = Fold-change, FFPE = Formalin-fixed, paraffin-embedded. (PDF) [file pone.0283159.s011.pdf]

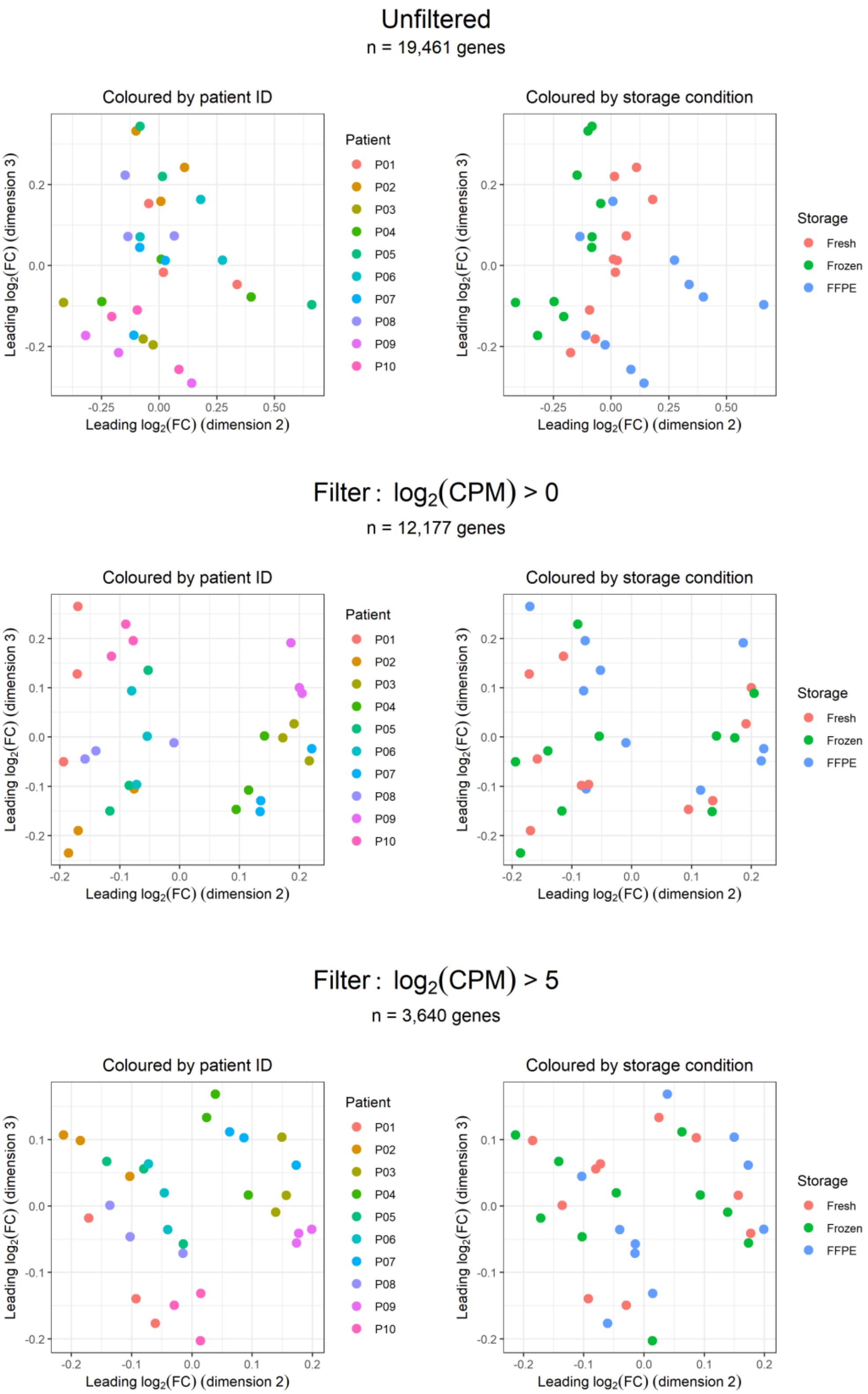

S11 Fig: Multidimensional scaling (MDS) plots (dimensions 2 and 3) of gene expression profiles of the unfiltered and filtered protein-coding RNA subset.

Abbreviations: CPM = Counts-per-million, FC = Fold-change, FFPE = Formalin-fixed, paraffin-embedded.
